# Diversity amongst Decision Makers? Workplace Inequality, Black Underrepresentation, and the Afterlife of Colonialism in NHS Governance

**DOI:** 10.17157/mat.11.2.7305

**Published:** 2024-04-29

**Authors:** Rebecca Irons

**Keywords:** NHS, Race and Ethnicity, Healthcare management, BAME, BME

## Abstract

Whereas senior management within NHS England was once so monocultural that it was dubbed the ‘snowy white peaks of the NHS’, recent data suggests that things have begun to change. However, Black staff in particular are still underrepresented. Interviews with Black and White NHS managers from four London trusts found that though the acronyms ‘BME’/‘BAME’ lack subtlety, management considered quantitative data important. The #BlackLivesMatter movement impacted NHS staff as a potential catalyst for change, though momentum fizzled out. Barriers to diversity and promotion have been seen to include microaggressions and negative stereotyping of Black staff. This article interrogates such underrepresentation, and uses the concept of ‘the afterlife of colonialism’ to suggest that NHS management hierarchies follow colonially-introduced hierarchies of ethnicity and discrimination, creating structural issues that are difficult to address. Taking a feminist framework of analysis, I will argue that diversity race work, including that of #BlackLivesMatter, is anti-hierarchical at its core, suggesting that it is difficult to assimilate within the hierarchically minded world of management. I will conclude that for ethnic diversity amongst staff to be realised, all staff must be committed to supporting this agenda, regardless of their race.

## Introduction

Senior management within the English National Health Service (NHS) was once so white-monocultural (Kline 2015) that Roger Kline coined the now oft-repeated phrase ‘snowy white peaks of the NHS’ as the title of his 2014 survey of racial inequalities in healthcare governance and leadership. At the time of Kline’s report, only 8% of London NHS trust board members were from a Black and Minority Ethnic (BME) background, with a mere 2.5% BME staff in positions of chief executive and chairs (Kline 2014, 3). That said, the numbers from the *Workforce Race Equality Standard 2021* (*WRES 2021*), a report that addresses ethnic inequalities in the NHS, look promising. Are these snowy peaks now melting? Addressing a woeful lack of qualitative data on the topic, this Research Article will explore the question in depth through discussing the experiences of racialised Black NHS senior non-clinical managers and their White counterparts working in London-based NHS trusts.

As I will discuss, managers expressed particular concern over the categorisation of ethnicity expressed through the acronyms ‘BME’/‘BAME’, though its utility for data collection and statistical triangulation was also acknowledged. Managers found that it could be difficult to replace, despite its contentiousness. #BlackLivesMatter (#BLM) was a significant topic of discussion, regarding both its presence and unrealised potential within the NHS, and staff describe how microaggressions and the absence of a pipeline of Black staff to move up the ranks of management negatively influence people’s career progression opportunities. This article will discuss the impact of #BLM alongside the perceived barriers to progression for Black staff within their trusts, to suggest that underlying structural racism constrains both the longevity and impact of movements such as #BLM as well as supporting stereotyping and discriminatory behaviours towards Black staff that impede their career progression. Utilising the concept of ‘the afterlife of colonialism’ (Gamlin, Gibbon, and Calestani 2021), I will discuss how colonial hierarchies of race continue to exist and influence institutions such as the NHS, leading to the experiences described by the interviewees.

As such, it will be possible to show how debates over the use of acronyms ‘BME’/‘BAME’ are fundamentally based in a critique of post-colonial, racialised hierarchies, which intersectional feminists suggest must be deconstructed in order to achieve racialised (and gendered) equalities. However, I consider that hierarchical structures are integral to management, making it difficult to address the present issues within the context of the NHS without also addressing the very structures of management itself. Instead, an initial and important step is for White management to ‘bother’ to empathise with Black colleagues, as suggested by Johanna Luttrell (2019). It is important to note that within feminist scholarship, empathy itself has been criticised when enacted by a White feminist towards a Black or Indigenous person residing in a settler colony (Lobb 2022). As such, Andrea Lobb advocates for a critical awareness of the workings of coloniality on the part of any would-be empathiser, otherwise this empathy runs the risk of contributing towards white supremacist ideals. From this critical perspective, it can be argued that only when the people in power are committed to dedicating their time and effort to equality within the workplace will it become within reach.

## Background

During the first years of the COVID-19 pandemic it was widely reported in popular media that BME people suffered higher COVID-19 mortality rates compared to White people in the UK. At the time, the UK parliament suggested that ‘underlying inequalities made the impact on some BAME groups far more severe than on their White counterparts’ (Women and Equalities Committee 2020, 3), though the government has since been accused of ‘“explaining away” Covid race issues’ and refusing to adequately address the underlying structural injustices highlighted by the pandemic (Gregory 2022). Public health expert Michael Marmot was reported as saying that the government’s denial of structural racism as a factor in unequal COVID-19 mortality relied on a ‘“misuse of evidence”‘ (Iacobucci 2021), which suggests a reluctance to face and deal with the consequences of embedded institutional racisms. Importantly, in the summer of 2020, alongside COVID-19 mortality reports came a reignition of #BLM, which re-erupted in protest and activism following the murder of George Floyd by police in Minneapolis, US in May 2020. With Floyd’s death thrusting #BLM to a new level of global awareness, ‘new outcomes and opportunities’ (Bhattacharyya et al. 2021) were created as a result—including, perhaps, within the NHS.

Indeed, official reports suggest that ethnic equality amongst NHS staff has begun to change. The introduction of the annual WRES in 2015 sought to address ethnic inequality in the NHS through reviewing data on career progression and discrimination. In the 2021 report (NHS England 2022a), the ‘total number of BME staff at very senior manager level has increased by 48.3% since 2018 from 201 to 298’, and ‘12.6% of board members in NHS trusts were from a BME background’, showing an improvement from the 10.0% reported in the 2020 WRES (4). ‘Very senior manager’ (VSM) level refers to ‘someone who holds an executive position on the board of an NHS trust or NHS foundation trust or someone who … holds a senior position typically reporting directly to the chief executive’ (Department of Health and Social Care 2021). These results have prompted NHS England to suggest that the ‘top jobs in [the] NHS’ are now ‘more diverse than any point in history’ (NHS England 2022b).

Though these percentage increases look to be a positive change, *WRES 2021* also highlights higher rates of discrimination and maltreatment of BME staff compared to White staff. For example, the report found that ‘the percentage of BME staff that personally experienced discrimination at work from a manager, team leader or other colleagues is at its highest level since 2015’ (NHS England 2022a, 26), with BME women in general management the ‘least likely to believe that their trust provides equal opportunities for career progression or promotion (54.1%), with low levels of belief amongst BME men in general management, too (63.5%)’ (25). Furthermore, BME staff were 1.14 times more likely to enter disciplinary procedures than White staff (Idem, 4), and still ‘remain underrepresented in senior positions’ (NHS England 2022b). It is perhaps unsurprising, then, that in a 2022 survey of BME leaders in the NHS published by the NHS Confederation’s BME Leadership Network, more than half of the staff responded that they had considered leaving the NHS due to racist treatment, particularly from other ‘colleagues, leaders and managers’ (BME Leadership Network 2022).

It is important to note that published data categorised under the acronym ‘BME’ is not disaggregated for the multiple different ethnicities to which it refers, and this is a key limitation when it comes to interpreting the information at hand. ‘BME’ is used ‘to define and sort many different ethnic groups by their shared characteristic of not being of white European descent’ (Gamlin, Gibbon, and Calestani 2021, 111), and therefore points to an exclusionary category of *non-White*. As such, statistics on BME staff do not provide an in-depth picture of the experiences of specific ethnic groups—for example, those of Black staff. This is a remarkable oversight, as the differences in seniority between Asian, Black, and White staff are quite pronounced. For example, data suggests that BME staff only made up 7.4% of VSM in 2021 (Cabinet Office 2021), however this percentage is not evenly split between the different ethnicities comprising the BME category. Data shows that 4.4% of VSM are Asian, with 1.3% Black and 92.6% White (Ibid.). Even when considered alongside the total number of Asian and Black staff employed by the NHS (10.7% Asian, 6.5% Black, 77.9% White), Black staff are still underrepresented compared to Asian staff at VSM level, with 91 out of 131,446 Asian staff at VSM level (0.07%) and only 28 out of 79,287 Black staff (0.03%). By contrast, 0.2% of White staff are at VSM level (Ibid.). This data is corroborated by the Nuffield Trust, who ‘investigated the proportion of people with black ethnicity across different NHS professions and compared this with the proportion of Black people in senior [pay] grades within these groups’, finding that the proportion of Black staff in senior pay grades (Band 7+) in each group is lower than the overall proportion of Black staff in that group (with the exception of midwifery) (Rolewicz and Spencer 2020). Nick Kituno and Lawrence Dunhill (2020) report that in 2020 at least 45% of NHS trusts in England did not have a Black staff member in a VSM position at all, suggesting that there are still issues surrounding diversity in NHS management, even if changes are slowly taking place. Indeed, *WRES 2021* suggests that Black staff report high levels of discrimination and a number of barriers to opportunity compared to other staff, with just 57.5% believing ‘their trust provides equal opportunities for career progression or promotion, with levels below those of other ethnic groups since at least 2016’ (NHS England 2022a, 24) and ‘19.4% of staff from a black background and 20.5% of black women in particular [having] experienced discrimination from other staff in [the] last 12 months’ (27).

The BME Leadership Network survey ascribed these ongoing inequalities to ‘structural and cultural issues … [which] led to a situation where BME leaders were not present in sufficient numbers to generate a climate of inclusivity’ (2022, 5). It is then safe to say that ‘White and BAME staff have very different and unequal experiences of the NHS as a workplace’ (Ross 2019).

Acknowledging the deeply embedded structural inequalities within the NHS, Gamlin, Gibbon and Calestani (2021) suggest that these inequalities are a product of colonial relations that saw the implementation and perpetuation of ethnic and racial hierarchies during the time of the British Empire. They refer to the ‘historically defined patterns and processes, along with the presence of colonial structures within the National Health Service itself, as the *afterlife of colonialism*, as they represent the permanence of the past in the present’ (108). Though Gamlin, Gibbon and Calestani did not invent this term (see Tharoor 2002; Cruz-Malavé and Manalansan 2002), their work is particularly relevant to the present discussion precisely because they link the NHS with colonialism and UK racial biopolitics to underscore how harm is brought to racialised communities due to the intersections of inequalities that exist in British society. Viewing structural inequalities within the NHS workforce as an afterlife of colonialism helps us to understand the differing hierarchies of management whereby White men still dominate senior roles (Ross 2019), even if changes are slowly taking place. Such racial hierarchies directly echo colonial relations of power, and their ongoing presence suggests that colonialism is indeed enjoying an afterlife when it comes to the control of power in the NHS. As the UK’s largest employer (Appleby 2018), structural inequalities may be present within the NHS, and indeed at any public institution existing in colonial afterlives, as Gamlin, Gibbon and Calestani contend. Developing these ideas further, I will apply an intersectional feminist framework of analysis to explore theoretical insights by Black feminists Angela Davis et al. (2022) that argue that all hierarchies, whether gendered or racialised, need be disassembled for equality to exist in society, with movements such as #BLM closely aligned to such thinking and approaches (Lebron 2023).

## Methods

The research upon which this article is based was funded by the Wellcome Trust and took place as part of a secondment fellowship at the Nuffield Trust. Research participants were all NHS non-clinical managers based in London and were recruited using existing networks, utilising my own contacts from previous research alongside NHS senior management in a large London trust, in addition to recruitment support from the Nuffield Trust and their existing network of contacts. As Daniel Souleles (2021) has problematised, when anthropologists ‘study up’ amongst those who hold and exercise power, a reconsideration of methodology is required. He suggests that this can be pursued through the use of methods other than participant observation, presence at multiple field sites, and the consideration of participants existing within networks of power, all of which the methodology in this project seeks to reflect.

As discussed above, the vast majority of managers in the NHS are White. As such, I had initially expected that the bulk of interviews would be with White managers. Though a number of respondents were White, the majority of my interviewees self-identified as Black, mixed-heritage, or did not self-identify. Beyond pure coincidence, this self-selection and volunteering to participate in such a study may reflect the unequal treatment towards Black people in the NHS, and therefore a greater eagerness to speak out about it and call out perpetrators.

I acknowledge that my own ethnicity may have acted as a limitation. As a White woman, I would not be able to share experiences of racism with Black interviewees. Despite this, I underscore the necessity of White researchers listening with critical empathy (Lobb 2022) and participating in the struggle for racial equality. Furthermore, within this study there was a complex dynamic of (in)equality at play between the participants and researcher that deserves further exploration. I am an ‘early career researcher’ and medical anthropologist, and interviewees held a considerably higher level of seniority than myself. Complicating Souleles’ (2021) exploration of ‘studying up’ further, this positioning meant that whilst participants and I shared a mutual recognition as professionals, other inequalities were clearly at play due to racial differences. This is likely to have influenced the research as it proceeded, with participants and I able to relate to each other on certain levels but not on others. I attempt to draw attention to this tension, where relevant, in the ethnographic vignettes and sections that follow.

I conducted 15 in-depth, semi-structured interviews with NHS management staff from four different London-based trusts. These interviews sought to understand the personal experiences, views, opinions, and reflections of NHS non-clinical management staff surrounding equality, diversity and inclusion (EDI) within their trust. London was chosen as it has the highest percentage of BME staff in the country, at 48.1% (NHS England 2022a, 10), and so in theory provided more opportunity for a wide range of experiences and reflections. Four interviewees self-identified as White, one chose not to give an ethnicity, one identified as mixed-heritage, and nine self-identified as Black. Inclusion criteria included staff of any ethnicity working in non-clinical management roles at band 7 and above.

Snowball sampling was used and participants were self-selected. Participants gave written consent. Interviews took place over Zoom between December 2021 and April 2022 and lasted approximately one hour each. Interviews were voice-recorded with the participants’ permission, and I later transcribed them. Transcriptions were then coded for recurring themes and analysed using anthropological frameworks of analysis including the afterlife of colonialism and feminist anti-hierarchical perspectives (Davis et al. 2022; Gamlin, Gibbon, and Calestani 2021). All participants and the trusts to which they belonged have been anonymised, with ethnicity (Black = B; White = W) and gender (M = Male; F = Female) identified for context related to comments in the results section.

This article reports upon interviewee views on both the terms ‘BME’ and ‘BAME’, and so I have opted to use both in my writing, whilst also acknowledging their limitations. Though the 2021 Commission on Race and Ethnic Disparities recommended that the UK government stop using aggregated terms such as ‘BAME’, as it is viewed as unhelpful and ‘demeaning to be categorised in relation to what we are not’ (Commission on Race and Ethnic Disparities 2021, 32), it should be noted that *WRES 2021* does use the term ‘BME’ (NHS England 2022a). As this article focuses on the NHS, its terminology will be used. It should be noted that though ‘BME’/‘BAME’ should not necessarily be used interchangeably, interviewees frequently did so, and so ‘BME’ is also retained in the analysis alongside ‘BAME’.

### #BlackLivesMatter: A catalyst to action

‘#BLM made a difference because people started to do some soul searching,’ one manager (WM) commented about the social movement’s influence on others within his trust and the wider community. Coinciding with the COVID-19 pandemic, the death of George Floyd and the visibility given to #BLM during the pandemic period was considered by the majority of managers with whom I spoke as a key and illuminative moment in the NHS, even if their opinions over any lasting influence differed.

One manager (BF) said that ‘a lot has happened since #BLM, people became more switched on, and we saw big-time changes’. This was because ‘when the pandemic hit, it was Blacks and ethnic minorities on the front line who were dealing face-to-face with patients getting sick, but the White colleagues weren’t working on the front line’. Another manager (WF) echoed this sentiment, saying that ‘definitely at the start [of the pandemic] the people dying were not White, not middle-class affluent doctors, and there was a realisation that there were not white faces on the front line’. However, when #BLM became more visible in the media, many of the interviewees commented that they saw a change within their trust. For example, one manager (BM) said that:

Since the George Floyd event people’s awareness has been awakened, though institutions could have a culture that perpetuates the disadvantages of Black and ethnic minorities, this was a moment to engage in that topic and start getting people to talk openly about it and change their thinking. Generally, this is what inspired the whole ‘let’s talk racism’ agenda that took place.

Another manager (BF) agreed that #BLM inspired open conversations, arguing that people ‘cannot be embarrassed to talk about these things’, and when #BLM became more visible, the Black community within her trust thought that ‘now it was our time to say things’. She continued, ‘#BLM has alerted people to everything that’s going on thanks to the media, now most trusts have recruited an EDI person or given them a bit more prominence in the organisation. There has been a difference, but it’s a shame that it has taken for a man to lose his life for #BLM to be taken seriously, this is quite sad.’

‘#BLM has been a catalyst of action’, suggested another manager (BM), as ‘the pandemic acted as both a pressure cooker and a magnifying glass’ to intensify existing attention on certain issues and put the spotlight on other, previously underexamined ones. Despite the pressures of the pandemic, another manager (WM) suggested that a focus on #BLM showed that ‘people do value diversity, this does matter to them’, and that related social events like ‘Prime Minister Boris Johnson refusing to take the knee and the use of derogatory terms like “woke idiots” contributed towards the public seeing things from a different perspective.’

However, whilst managers recognised that #BLM did raise the visibility of ethnic inequalities in healthcare (and beyond) within their trusts, not everyone was convinced that this would contribute towards any lasting changes within the NHS. As one manager (BM) said, ‘#BLM at its peak made people in the organisation feel like they needed to do better than now, but this all fizzled out. Some took more notice and wanted to make it look like they were doing more, like all of a sudden, a Black voice appeared in the trust, but now it’s fizzled out into nothing again.’

Similarly, a manager (BF) reflected that ‘#BLM created a whole thing where people before had been scared to stand up and say anything, now they had big campaign posters for stamping out racism and everyone had a poster to support #BLM … everyone took the knee and held a poster.’ However, she noted that not everyone seemed happy to support these initiatives, as ‘when we had quite a challenging and important moment putting up a poster for #BLM, I passed a White colleague on the stairs and they just stood there eating their sandwich in the middle of this moment, so people were obviously uncomfortable.’ She continued that ‘it’s not just about George Floyd, you need to look at the bigger picture, some got lost with just taking the knee for George, some people were criticising Black people for supporting a man who didn’t lead the best life, everyone was missing the point regarding what he had done, people didn’t get it.’ The manager explained how her family had been affected by incidents of police discriminatory behaviours and that this was the point of #BLM, but that White colleagues often misunderstood this and wrongly interpreted #BLM as a protest solely against the death of one man. ‘People just didn’t get the concept’, she said, ‘this day in, day out, taking the knee … the NHS didn’t understand what it was about, or they just thought it was a moment in time. And after that, no more reactions.’

### Barriers to progression for Black staff

‘What’s the biggest barrier [for Black staff to progress to senior management roles]? Well, it’s people’, one manager (BM) suggests. ‘This is very difficult territory, the topic of racial discrimination. Everyone within the NHS believes themselves not to be [a racist], no one says, “yes, I’m racist,” and it takes quite a bit of courage to call out others.’ He noted that speaking about race and racism is ‘highly taboo, difficult, and at the uncomfortable end of the spectrum’, and ‘that’s a huge barrier.’ ‘Why would you even want to go there?’ he pointed out, when staff are likely to ‘not get any recognition, but most likely a load of hassle’; he found it unsurprising that much-needed conversations around race, ethnicity, and diversity in the NHS were simply not taking place. He remarked that in his trust, racism was mostly covert and easily deniable (though not all managers felt this to be the case in theirs). He felt that ‘it would be much more proactive for White staff to say we need to change the way we approach this [inequality]’, rather than relying on Black and minority ethnicity staff to do so.

One reason for this is that White staff are ‘more likely to have the ear of the chief executive’, as another manager (BM) also noted. As such, there was ‘no [Black] representation at [senior management] meetings and they can just say what they want. Management listens to other [White] colleagues and we [Black staff] haven’t got a look in.’ The dearth of Black senior management was mentioned by many of the interviewees, and was frequently highlighted as one of the key barriers to ensuring the career progression and promotion of Black staff. For example, one manager (BM) said:

The access to leadership of the trust does not reflect the composition of the staff, the Black and minorities are literally not present—there is no Black director, no Black person on the executive board, the Black staff literally do not feel that they have anybody up there representative of them who could be trusted in confidence, and who reflects their aspirations.

He argued further that this was not simply an issue of representation, but reflected a deeper cultural barrier for Black staff: ‘people feel that leadership may never understand their concerns, as you’re dealing with different kinds of cultures … it is not always because of malice, but culturally they [White management] don’t understand when someone from a different cultural background expresses themselves in a particular way.’ He said that these cultural differences extended to talent: ‘the way that talent is identified is deeply rooted in unique cultural understandings of talent’.

Other staff felt that barriers to diversity were as a result of unconscious bias. For example, one manager (BF) spoke about her experiences working on Zoom during the pandemic, and how she was the subject of microaggressions. She mentioned that when she is sending emails to colleagues who have not met her personally, they do not know she is Black (her name did not indicate her ethnicity). However, she said that when she joined Zoom meetings, her White colleagues would ‘go, “Oh!”‘ in surprise, and she believes this is because they were not expecting her, given her senior position, to be a Black woman. Similarly, she noted that when she used to go to in-person conferences as an NHS representative, she would often raise her hand and speak to the room. She felt her English accent may be perceived as ‘posh’, and she remembered how ‘people would be looking around the room for another person, because they didn’t expect a Black woman to speak like that.’ She felt that her initial relationships with White colleagues can be a ‘bit frosty’, and that such microaggressions acted as barriers to progression and diversity for Black staff. This woman’s experiences reflect Nirmal Puwar’s notion of ‘space invaders’ (2004), whereby (gendered) racialised individuals are not entitled to the same institutionalised spaces as others (White, male). In particular, Puwar challenges the idea that professional positions are ‘colourless’, showing how the opportunities for advancement and flourishing upon receiving a job are less available for those racialised as Other (2004, 55). The surprise expressed by the quoted Black manager’s White colleagues at seeing her in the managerial space suggests that she may have been perceived as invading the (White, male) space where, by extension, she was unwelcome.

Another Black female manager had similar experiences and concerns regarding interactions on Zoom with majority-White colleagues. She said that the lack of Black representation in senior management ‘influenced my willingness to participate in meetings, I didn’t want to put the camera on as I know how they might feel, I was nervous to speak up and I needed to find my voice’. As she got to know them better she became more comfortable interacting with her White colleagues, but she still felt like she needed to make more effort in her role than White counterparts: ‘I always have to be on my game, others can drop the ball but I have to give 120% all the time, I always have to give more effort’. Though she worked in a senior management role, she said there was no ‘pipeline of Black staff’ that might follow her and aspire to a senior role. She mentioned that she did not always feel able to speak up in case she was perceived as confrontational. She believed that this was a principal barrier to more diverse senior management teams. A different Black female manager mentioned turning off her camera on Zoom calls, as she ‘felt like a dot in the ocean of Whiteness’ and did not want to appear on the screen.

One Black male manager spoke about his experiences of being demoted due to the perception of a White female senior manager of him as ‘aggressive’ following a workplace dispute. He noted that he felt ‘voiceless’ after this incident, as he did not consider his behaviour to have been aggressive despite being told by the ‘firing committee’ that his ‘style and tone’ were so. When discussing barriers to diversity, he mentioned that ‘it’s pretty obvious they [White management] don’t want to share the spoils. Even with simple things like appraisals, these reminders flash up and management just overlook it, so you don’t get a chance’.

All that said, many trusts are attempting to address diversity in their management teams by using ‘diverse’ interview panels, a strategy employed by all of the trusts from which I interviewed staff. Such panels require that at least one interviewer must be from a self-identified Black or minority ethnic background. These panels had mixed reviews amongst interviewees. One manager (BF) said that ‘people just interview their friends’, thereby perpetuating the cycle of discrimination and creating a barrier to diversity. Another manager (BM) suggested that management jobs are not advertised equitably, creating a set of issues around ensuring a pipeline of Black staff to progress through management roles. He argued that ‘we recruit from the human race … as racial prejudices predominate in the societies in which we live, therefore they exist in who we recruit.’ He went on to suggest that ‘the biggest barrier is that we don’t yet have the people who want to implement change. We wouldn’t be having this conversation if there were a group of people in the trust for whom EDI were an important thing.’ I have known this particular manager for a number of years, have worked with him previously, and we have had multiple conversations together about EDI in the NHS. His comment references the notion that, though anthropological enquiry into racial inequalities in the NHS may be valuable, it perhaps *shouldn’t* be necessary. The very fact that an external researcher has had to plan, seek funding for, and execute a project on NHS management diversity, constituting one of the only reports into Black experiences within the organisation, speaks volumes.

The contentious stereotype of Black people acting hostile at work, and particularly that of ‘the angry Black woman’, abounds, and affects people’s ability to progress and enjoy their work life (Motro et al. 2022). However, speaking up and acting assertively are perceived as key and highly desirable skills for managers to possess (Santora 2007). If Black staff display the kinds of personal qualities that are desirable in White management staff, they may face discrimination or barriers to opportunity. These are clearly instances of racism, and so, as Christopher Lebron observes, Black people must continually do the double work of seeing themselves through White eyes (2023, 138). However, overcoming these issues will require more than addressing individual experiences. These are structural problems of institutionalised racism expressive of the ‘afterlife of colonialism’, and suggest that though inequalities and discriminatory behaviours are no longer officially institutionalised, as during colonialism, they now continue in the background, unchecked, in the NHS and elsewhere.

### Equality, Diversity, and Inclusion: A ‘Holy Trinity’

Closely related to ethnic inequalities and the intent to address these is the acronym ‘EDI’—not least because some of the managers I interviewed were ‘EDI leads’ within their trusts. Indeed, ‘EDI’ has become somewhat of a buzzword in the NHS and beyond, and the three terms it acronymises have been referred to as a ‘holy Trinity’ (Köllen, Kakkuri-Knuuttila, and Bendl 2018) in organisations and businesses.

Though their responses to EDI differed, there are common themes that characterise the interviewed managers’ reflections on the use and utility of the term, surrounding its translation to statistics and data, and the seriousness with which it is taken. For example, in three of the managers’ trusts the EDI leads were also involved with in-house staff networks related to ethnic equalities and supported the development and promotion of specific activities related to celebrating diversity. An example of this in two trusts were the celebrations of Black History Month, including a book club and invitations to external speakers to lead sessions on cultural change. Though such initiatives were praised by interviewees, it was noted that there was some resistance amongst other members of staff. For example, a manager (BF) recounted how, upon receiving an email about the Black History Month Book Club, a White colleague turned to ask her ‘have you seen that fucking meeting on Black history month?’ She noted that this person did not value the importance of discussing race and clearly just ‘didn’t get it’ due to their White privilege, seeing the meeting as a hassle. Other managers mentioned similar incidents that arose when senior management was asked to engage in EDI endeavours. For example, another manager (WM) said that ‘senior managers start to do their emails whenever EDI comes up in meetings’. Though discouraging, some insight was provided as to why senior management disengage with EDI.

One manager (WM) explained how EDI is used often in the ‘language of organisational development’, and is increasingly employed in the strategy and forward planning of his trust, as exemplified by their recent hire of a new ‘EDI manager’. However, he suggested that the current EDI work was not necessarily successful at holding the attention of senior management as it took a ‘flowery, soft approach’ that did not ‘take the audience into account’. He argued that ‘if you want to appeal to managers you need to give them an action plan with hard targets and deadlines … you need to engage the people you want to respond with’. The present EDI strategy in his trust was ‘very woolly’ and did not fit well with managerial frameworks of thought and understanding, which were much more based on quantifiable outputs and results. This manager argued that he considered it important to make things relevant to managers if one wanted to engage them with issues outside of their ‘core’ role—such as EDI work. Another manager (WF) concurred that EDI was seen as a ‘soft attempt to celebrate difference and culture’, and was not impactful amongst senior management, with another (WM) calling it a ‘flaky’ term. One such issue with EDI work that was highlighted was that, in its current form in the trusts from which staff was interviewed, it mostly took place in the form of optional events and communities but did not produce significant statistical data about change (for example, by reporting actual, lived experiences of racialised management staff, as detailed in this paper, and/or survey results about cultural change that could take place). Though this may be an oversimplification of what matters to senior management, one manager (BM) proposed that ‘[hard] data is the most useful drive in taking decision-making action into interventions’, and EDI work was not producing this data at present. That said, the other acronyms discussed widely in interviews were very much related to data collection (and therefore decision making), which complicated managers’ opinions of them and perceptions of their utility.

### The BME/BAME acronyms in the workplace

When it comes to acronyms referring to ethnic and diversity terms, the ethnic-identifier acronyms ‘BME’ and ‘BAME’ are perhaps the most contentious and were widely discussed in the interviews for that reason. An overarching reaction was that the terms ‘BME’/‘BAME’ did not accurately convey the differences between the groups of people to whom they refer. ‘BME lacks subtlety’, one manager (WF) commented; another (WM) called it an ‘ambiguous term’. For one manager (WM), the acronym was particularly negative. He said that he ‘hated’ the term. Now, BME/BAME ‘is just a clunky way of categorising race so that we can label something that is complex, people say everyone is dark so put the same label’.

Another manager (BF) saw the acronym as just a ‘tick-box’ that may or may not be used against a racialised person. For example, she stated how her trust has organised a senior leadership program aimed at staff who self-identify as BME, which could be a good thing, or just a way for the trust to show they are ticking a ‘diversity box’ without implementing any real changes. ‘There are varying views amongst the BME community itself’, she observed. The perception that the acronym had both positive and negative qualities was shared by another manager (BF), who said that it did help create ‘a group awareness of what are they doing for the community’ but that on the other hand it ‘leaves out those who are Asian, or Eastern European’ as it tends to refer to Black people. She continued, ‘what is Black? Being Black, there are many different types of Black, in terms of socio-economic status, ethnic mixture, different countries of origin … everyone gets sucked into one terminology’. That said, she noted that even though diversity and distinction may exist amongst communities, ‘a policeman won’t ask what’s your race, there is just a skin colour perception’, so perhaps a focus on ‘BME’ was needed. The perception that the acronyms ‘BME’/‘BAME’ mainly referred to Black individuals was shared by other managers as well, though with differing ideas about whether this was positive or not. For example, one manager (WM) said that though it was widely known to refer to ‘minority ethnicities’, everyone always emphasised the focus on Black people, and another (BM) thought that the use of this acronym was intentionally exclusionary towards Asians and other ethnic minorities. He said that the ‘BAME acronym mainly focuses on Black colour skin, so it has not been helpful in the sense of inclusion. Experience has shown that the acronym has become reduced to Black coloured skin, and this is not helpful.’

From another perspective, one manager (BF) agreed that the term was exclusionary, but that this was desirable. As she mentioned, Black people have been subject to discriminatory behaviours and racism across history and were still underrepresented within NHS senior management and clinicians, even when compared to other ethnicities. As such, she said, ‘you don’t want to lose the focus on race, we don’t want to dilute the focus to be on all ethnic minorities, we need to keep the focus on Black people.’

Another view about the utility of the term was inspired not by its semantics, but the fact that it enabled a collection of data that made sense within the organisation. For example, one manager (BM) argued that ‘it is useful, and though there is talk about changes this isn’t a good idea—all the statistics have been based on BAME so it has muddied the water, but you would leave it muddier [if you changed it]. There are lots of different terms and no matter which you use there will be someone who is unhappy with it.’

On a similar note, another (WM) said that ‘from a policy point of view the shorthand has a utility, and we can recognise a lack of equality this way’. Though it may group people together somewhat broadly, the acronym acted as a ‘general shorthand way of talking about things’, and importantly, this is key within the world of management.

Summarising the arguments, one manager (BM) said,

Everyone has a view about the helpfulness of lumping people together [under BME/BAME], and after the government review [Commission on Race and Ethnic Disparities 2021] recommended that BAME no longer be used the trust felt it was obliged to change policy, but there were still a variety of views. Not least, as many were angry about the report that suggested there was no such thing as institutional racism in the NHS, which was a deliberate act of provocation.

However, at his trust’s ethnicity-staff network meeting it was decided not to remove the acronym from their group working title.

As such, though the ‘BME’/‘BAME’ acronyms are contested as being too general and ambiguous resulting in exclusions, there were still those who thought it had merit and utility for reasons ranging from the statistical data it is used within, to a much-needed focus on Black staff within the NHS. As mentioned in the previous section, EDI was not contested directly, though it was described using words like ‘woolly’, ‘flaky’, and ‘flowery’ by interviewees, which may suggest it is not viewed as a category that can usefully contribute to statistical data sets as BME/BAME do. Nevertheless, the two do go hand in hand and are often discussed together.

The fact that the acronyms ‘BME’/’BAME’ are viewed as failing to adequately project the vast differences and ‘subtleties’ between the groups of people that they seek to represent is not a new finding. The acronyms were recommended for removal from government documents in the Commission on Race and Ethnic Disparities (2021) precisely for this reason, and Gamlin, Gibbon and Calestani suggest that they disguise ‘the role of [racist] social and political history’ (2021, 111), thereby contributing to an afterlife of colonialism. One manager (WM) did indeed note how the history of plantation work in Jamaica during colonialism produced a ‘hatred on that island [Jamaica] between races’, with the acronyms ‘BME’/‘BAME’ serving as a division marker rather than addressing that hatred.

Indeed, on obscuring subtleties through acronym use, business anthropologist Gillian Tett notes, ‘if you want to hide something in the twenty-first century world, you don’t need to create a James Bond-style plot. Just cover it in acronyms’ (2021, 93). ‘BME’/‘BAME’ do obscure data on racial inequalities, for example by failing to highlight the ongoing differences in seniority between Asian and Black employees, as mentioned in the introduction. Nevertheless, an important point is that these acronyms do actually allow for the collection and analysis of quantitative data sets. As the interviewed managers commented, ‘the shorthand has a utility’ for policy, and it might muddy the statistical waters even further to disaggregate the data and do away with the acronyms now. But perhaps even more significantly, these acronyms and the data sets that can be produced with them respond to the expectations of senior management. As one interviewee noted, it is important to engage the target audience, and the ability to represent inequality in reports such as the WRES is important to the audience in question.

## Conclusion

That colonial hierarchies of race continue to produce inequalities in contemporary Britain is a realisation that increasing numbers of individuals are coming to, exemplified not least by movements such as #BLM and the removal of slave trader Edward Colston’s statue in Bristol in 2020, for example. However, decolonial struggles are enormous undertakings, and whilst it would be reasonable to conclude that a total deconstruction of racialised hierarchy is what is truly needed for lasting change in institutions like the NHS and beyond, the enormity of this task must also be recognised. Furthermore, the potential conflict between management structures of hierarchy and the idea of deconstructing workplace hierarchy will undoubtedly pose a barrier. Strides have been made in terms of gender equality in the workplace, and as the data presented in the introduction showed, so too are changes slowly taking place regarding race inequality in the NHS. However, as feminist scholars Davis et al. (2022) and others may argue, for as long as any institutionalised hierarchies exist, genuine equality will be difficult to achieve. This article does not suggest that the NHS abolish its structure of management hierarchy, as I acknowledge that this may not seem a practical (or desirable) endeavour at present, even if ultimately this may prove the solution to addressing the inequalities in the workforce. However, it is necessary again to reflect on the work of Davis (1974) and her Marxist perspective to understand the harm that hierarchies perpetuate in society.

Management is all about the delineation of, and adherence to, hierarchy. That is not a revelation, as indeed there are levels of seniority that the NHS itself makes expressly clear through, for example, its pay band hierarchy. However, race work is about the *disruption* of racialised hierarchy, with #BLM directly implemented in this. I am suggesting that the important work of addressing and deconstructing (racialised) hierarchy inevitably finds resistance in a context where hierarchy is valued, upheld, and important (the fact that those at the top of the hierarchy are White males is important, but not the key point here). As Paul Gilroy notes, anti-racism become reduced to ‘empty, ethereal statements’ when ‘trivialised in the poetry of management science’ (2002, xxx). If the NHS wishes to address racial inequalities amongst staff, it will also need to contend with ingrained structures of hierarchy, and this is difficult in a management context. For example, as Emma Dabiri (2021) argues, racist structures of inequality are supported and upheld by other unequal structures that disadvantage some for the benefit of others. She suggests that capitalism is one of those structures, due to the exploitation of workers for the benefit of the privileged few. However, it is difficult to imagine a management structure outside of capitalism as it is deeply related to this economic model. In this context, an intersectional feminist framework of analysis may be instructive.

Radical feminism has long upheld an anti-hierarchical position, taking the view that (gendered) power is institutionalised and so the only way to disrupt (gendered) inequalities is through the deconstruction of the institutions that uphold and support them (Voigt 1990, 26–27). Though early feminist theorists failed to acknowledge the compounded struggles of non-White women, Black feminist Angela Davis (along with others) has developed an intersectional perspective to argue that gendered *and* racialised hierarchies operate together to create and reinforce inequalities, leading to the suggestion that abolition is the solution to overcome institutionalised, post-colonial systems of oppression (Davis et al. 2022). Black Panther member Davis’ long-standing work is as a Marxist feminist, and she is a strong advocate of prison abolition (1974). Davis views imprisonment as related to one’s gender, race, class and sexuality above and beyond the crime one has committed (Ibid.). Though this might not seem immediately relevant to the NHS, Davis’ work does develop important ideas surrounding institutionalised racism that merit further attention beyond prison reform. All hierarchies serve as a basis for inequality and potential discrimination. This article focuses on racial hierarchies within one organisation, but one must recognise that inequalities intersect across race, gender, class, and sexuality as well. Viewed from a Marxist perspective, the only way to address inequalities in society is to deconstruct the hierarchies that uphold them. Ultimately, where ideological hierarchies exist, whether gendered, racialised, or both, it will be difficult to truly obtain equality. Davis et al. (2022) argues that state institutions built on the backs of colonialism (such as the prison system, particularly in the US) should be abolished altogether as they will forever be unable to change what is fundamentally a racist institutional structure.

This is not to suggest that the NHS be abolished, but that intersectional feminist thought is a relevant and important framework of analysis to think through the institution’s underlying structural issues. The deconstruction of racist hierarchies goes hand in hand with the deconstruction of other ideological hierarchies, including that of gender, and extending even to human/non-human hierarchies for some feminists such as Haraway (2016). Ultimately, EDI work in the NHS could come into conflict with hierarchy itself, as it seeks to remove ideological barriers of superiority and inferiority. The hierarchical nature of management structures could occasion an ideological clash along these lines. This tension may offer explanation as to why the managers interviewed found that the flames that #BLM initially sparked soon fizzled out—the anti-hierarchical movement (Lebron 2023) does not fit ideologically with management structures of hierarchy.

At its heart, EDI work in a given institution is about care and caring for others. Feminists have long recognised that care is feminised, and it is not a surprise that people associate caring work with ‘soft’ adjectives that are often gendered female (Towns 2020). However, management and leadership are ‘heavily masculinist in culture’, including for female managers who embody masculine discourses to succeed in the workplace (Whitehead 2014, 438). This suggests a potential incongruency between how management is viewed and views itself, and where EDI fits in. This is relevant to the discussion surrounding EDI, as EDI work is seen as feminised by managers, and therefore in contrast to, and potentially unwelcome in, the masculinised world of management. But EDI work is all about shining a light on inequalities and addressing racialised hierarchies. If it is not seen as congruent within the world of management—and indeed the stories that the interviewees mentioned, such as senior management turning to do their emails whenever EDI comes up in conversation during meetings, would suggest it is not—then perhaps the task of addressing racialised hierarchies is also incongruent with management structures as they currently stand.

This article has argued that hierarchies must be dismantled for greater equality to exist amongst the NHS workforce in England, whilst also recognising that managerialism explicitly relies on hierarchies, which makes this seem an impossible endeavour. Yet, prior to the reforms of the 1980s, the NHS was a socialist institution that held quite different values from the business model under which it increasingly operates nowadays (Mulholland 2009). As such, in theory it is possible for an organisation like the NHS to follow a Marxist logic of flattened hierarchy, whereby no manager is placed in a position of seniority and power above others, if it were to abandon its neoliberal business model (which is unlikely).

Instead, I suggest that the first task of import is for NHS management staff, and particularly White staff, to begin to pay attention and give their time to important EDI work that seeks to make changes within the organisation. This article has explored how some managers do not dedicate attention to this kind of work, and how it is framed and described in a way that feminises it, placing it ideologically outside of the masculinist world of management. But it is important that this change. As Luttrell argues, the first step towards racial equality is for White people to believe that the effort required is worth their time (2019, 7). One manager noted that [White] managers were unlikely to bother with EDI work because of the hassle, but as Dabiri argues (2021), White people must begin to abandon feelings of guilt and take up the ‘hassle’ to empathise. This is a task that is wholly possible within the scope of current NHS management, and one that should be prioritised.
